# Burnout among Nurses Working in Ethiopia

**DOI:** 10.1155/2020/8814557

**Published:** 2020-10-16

**Authors:** Abrha Hailay, Woldu Aberhe, Guesh Mebrahtom, Kidane Zereabruk, Guesh Gebreayezgi, Teklehaimanot Haile

**Affiliations:** ^1^Department of Adult Health Nursing, School of Nursing, Aksum University, Aksum, Ethiopia; ^2^Department of Epidemiology, School of Public Health, Aksum University, Aksum, Ethiopia; ^3^Department of Maternity Nursing, School of Nursing, Aksum University, Aksum, Ethiopia

## Abstract

**Background:**

Burnout is a condition of emotional exhaustion, depersonalization, and low personal accomplishment that can occur among individuals who work with people in some capacity. Nursing is a stressful profession that deals with human aspects of health and illness and can ultimately lead to job dissatisfaction and burnout. Although burnout among nurses has been addressed in previous research, the heterogeneous nature of the result findings highlights the need for a detailed meta-analysis in Ethiopia. Thus, this review is aimed at identifying the prevalence of burnout among nurses in Ethiopia.

**Methods:**

A search strategy was implemented using electronic databases (PubMed/MEDLINE, Google Scholar, Web of Science, Cochrane Library, Africa-Wide Information, and African Index Medicus) which were systematically searched online to retrieve related articles using keywords. Studies which were included in this review were written in the English language because writing articles in other languages in Ethiopia is uncommon. The combination of key terms including “burnout”, “nurse” and “Ethiopia”, “systematic review” and protocols was used. The Preferred Reporting Items for Systematic Reviews and Meta-Analysis checklist guideline was followed stepwise. All published articles starting from inception to February 2020 were included, and we did not find unpublished studies. Heterogeneity across the included studies was evaluated by the inconsistency index. All statistical analysis was done using R and RStudio software for Windows, and a random-effects model was applied to estimate the overall prevalence of burnout among nurses in Ethiopia. It is registered in PROSPERO (CRD42020188092).

**Results:**

The database searched produced 1060 papers. After adjustment for duplicates and inclusion and exclusion criteria, seven articles with 1654 total nurses were found suitable for the review. Except for one cohort study, all studies were cross-sectional. The overall pooled prevalence of burnout among Ethiopian nurses was estimated to be 39% (95% CI: 27%-50%).

**Conclusions:**

Burnout affects two out of five nurses in Ethiopia. Therefore, effective interventions and strategies are required to reduce burnout among nurses.

## 1. Background

Nursing is a stressful profession that deals with human aspects of health and illness and can ultimately lead to job dissatisfaction and burnout. The profession is not only physically demanding while dealing with human health and function but also necessitates the use of mental energy and leads to mental exhaustion when one is continuously exposed to stressful events and situations. This mental exhaustion is what partly defines burnout [1, 2].

Burnout is a condition of emotional exhaustion, depersonalization, and low personal accomplishment that can occur among individuals who work with people in some capacity [3]. The emotional exhaustion component is characterized by loss of emotional resources and energy, lack of enthusiasm, frustration, tension, and fatigue. The depersonalization component represents the interpersonal relationships that lead to a negative interaction. The sense of low personal accomplishment refers to feelings of incompetence [4].

Most of the time, burnout can occur due to the presence of job demands like work overload, prolonged working hours, nurse-patient ratio imbalance, role conflict, lack of fairness, conflict in values and job resources like lack of social support from colleagues or management, lack of feedback, and poor participation in decision-making [5]. It represents a high cost to workers and their institutions and appears to be more common in developing than in developed countries [6].

WHO recently declared burnout as an “occupational phenomenon” in the International Classification of Diseases 11th revision (ICD-11), recognizing burnout as a serious health issue worldwide [7]. Other evidence also suggested that burnout in nurses is high across specialties and countries [8]. Globally, the overall prevalence of burnout among nurses is 11.23% but significant differences were noted between geographical regions and specialties. The sub-Saharan African region had the highest burnout symptom prevalence rate while Europe and Central Asia region had the lowest [7]. A systematic review conducted in sub-Saharan Africa showed that the prevalence of burnout among nurses was 33% [9].

Some of the consequences of job burnout were absenteeism, low morale or personnel deterioration, stress, anxiety, psychosomatic complaints, sleep disturbance, and poor organizational commitment [5]. Burnout not only affects physical and mental abilities but also affects the individual's health [10, 11]. Therefore, identification and prevention of burnout play an important role in improving the quality of provided services [12].

Ethiopia's health system is among the least developed in sub-Saharan Africa, and it is not able to cope effectively with such massive health problems; nurses are very poorly paid, and their working conditions are demoralizing which is often unsafe as they strive to provide services with few supplies and equipment that leads to burnout. According to the Ethiopian Nursing Association (ENA), about 43,500 nurses are working in the government system and the ratio of nurses to the population is about one nurse for 2299 people [13].

In Ethiopia, the prevalence of burnout among nurses has been addressed in previous research but the reported prevalence of burnout among the studies was heterogeneous which ranges from 12% to 50% [14–17]. Therefore, this finding highlights the need for a detailed meta-analysis in the nation Ethiopia. Thus, this review is aimed at determining the pooled prevalence of burnout among nurses in Ethiopia. The potential benefits of this study are for nursing professionals, nursing practice, nursing managers, decision-makers, and researchers. The research question for this review was what is the prevalence of burnout among nurses in Ethiopia?

## 2. Methods

This systematic review was performed according to burnout among nurses in Ethiopia using a priori protocol registered in PROSPERO (CRD42020188092).

### 2.1. Study Setting

Ethiopia is one of the East African countries and has 9 regions, namely, Tigray, Afar, Amhara, Oromia, Somali, Benishangul-Gumuz, SNNPR, Gambella, Harari, and two administrative states (Addis Ababa city administration and Dire Dawa city administration). According to the Ethiopian Nursing Association (ENA), about 43,500 nurses are working in the government system and the ratio of nurses to the population is about one nurse for 2299 people [13].

### 2.2. Search Strategy and Information Sources

A search strategy was implemented using electronic databases (PubMed/MEDLINE, Google Scholar, Web of Science, Cochrane Library, Africa-Wide Information, and African Index Medicus) which were systematically searched online to retrieve related articles using keywords. Studies which were included in this review were written in the English language because writing articles in other languages in Ethiopia is uncommon. The presence of precursor systematic review and/or protocol on the topic of interest was checked via searching different databases for the systematic review. The databases searched include the Cochrane database of a systematic review, Joanna Briggs Institute database of a systematic review and implementation reports (JBI-DSRIR), the national health center review and dissemination database, health technology assessment (HTA), the Campbell collaboration library, and evidence for policy and practice information (EPPI-Centre).

The literature search technique was developed using the headings of the medical subject headings (Met); BOOLEAN (AND/OR) operator was used. The combination of key terms including “Burnout”, “Nurse” and “Ethiopia”, “systematic review” and protocols was used. The search from the above databases confirmed that there was no systematic review and/or protocol on the topic of interest.

### 2.3. Data Selection, Extraction, and Process

The two reviewers screened the titles, abstracts of all citations retrieved, and the full-text search results to identify potentially eligible studies. Where necessary, authors were contacted for additional information to confirm eligibility of studies. Disagreements were resolved by discussion after mutual consensus and a third independent reviewer.

Data extraction used a data collection form and was performed by two blind and independent reviewers. Where there is missing information, the corresponding author of the study was contacted to request the missing information. An email was sent to the corresponding author to request for additional information before excluding the study. For studies appearing in more than one published article, we considered the most recent, comprehensive, and with the largest sample size. For surveys appearing in one article with multiple surveys conducted at different time points, we have treated each survey as a separate study. Data extracted format comprised information about the year of publication, data collection period, region, sample size, design of the study, diagnostic criteria of burnout, and prevalence of burnout.

### 2.4. Criteria for Considering Studies for the Review

#### 2.4.1. Eligibility Criteria (Inclusion Criteria)

*Design*: all published observational studies (six cross-sectional and one cohort studies) were included.

*Population*: studies which included nurses and reported independently (the included studies were conducted in general nurses without specification of units/ward) were taken as part of the analysis.

*Publication status*: peer-reviewed articles were included in this systematic review and meta-analysis.

*Settings*: all health institution-based studies were included.

*Language*: articles published in the English language were included in this review.

*Publication or report year*: all published articles starting from inception to February 2020 were included.

*Method of diagnosis*: all of the studies which used the English version of Maslach's Burnout Inventory-Human Services Survey (MBI-HSS) to assess burnout were included.

*Outcome*: prevalence of burnout among nurses was the outcome of the study.

#### 2.4.2. Exclusion Criteria

We excluded studies without sufficient statistical information for performing meta-analysis calculations and studies with data from other professions but without independent information for nurses. Additionally, a study that did not use Maslach's Burnout Inventory-Human Services Survey (MBI-HSS) for diagnosis of burnout was excluded from the review because of uniformity of the measurement and to use standardized measurement.

### 2.5. Quality Assessment and Risk of Bias in Individual Studies

A tool developed by Hoy et al. for prevalence studies was used to evaluate the likelihood of bias and quality of studies included in this review [18]. The tool contains 11 items; items 1–4 assess the external validity, 5–10 assess the internal validity, and item 11 offers a description of the overall risk by the reviewer based on the responses of the above 10 items which are rated 1 if yes and 0 if no. Studies are graded as low (<3), moderate risk (4-6), or high (7-9) risk of bias. Two reviewers did this exercise, and the disagreements were solved by one experienced reviewer in systematic review and meta-analysis. Moreover, adequate sampling methods, consistent methods and procedures for collecting data, recorded methods of quality control, and representative sample size were considered as indicators of the study quality. The methodological quality of the included studies was evaluated using the Newcastle-Ottawa Scale for cross-sectional and cohort study. The Newcastle-Ottawa Scale was designed to assess the quality of nonrandomized studies in meta-analyses. This scale is primarily formulated by a star allocation system, assigning a maximum of 10 stars for the risk of bias in three areas: a selection of study groups (4 or 5 stars), comparability of groups (2 stars), and ascertainment of the outcome of interest or the exposure (3 stars). No validation study provides a cut-off score for rating low-quality studies; a priori, we arbitrarily established that 0–3, 4–6, and 7–10 stars would be considered at high, moderate, and low risk of bias, respectively [19]. No study was excluded because of methodological bias because six of the studies were highly qualified while one study was moderately qualified based on the Newcastle-Ottawa Scale. Therefore, all of the studies were included in this review.

### 2.6. Data Management

Based on the inclusion and exclusion criteria, a tool has been developed a priori to guide the screening and selection process. The tool was piloted and revised before data extraction begins. The search results were uploaded to EndNote software first to remove duplicates, and 391 studies were removed due to duplication.

### 2.7. Data Items (Data Collection)

We included the following data from each study in this meta-analysis: data on general information, authors, year of data collection and publication, the region where the study was performed, assessment method of burnout, type of publication, study design, setting, total sample size of nurses, and total cases with burnout.

### 2.8. Outcomes and Prioritization

Prevalence of burnout among nurses: to assess the prevalence of nurses' burnout, studies which used Maslach's Burnout Inventory-Human Services Survey (MBI-HSS) were used, which comprises 22 items with 8 items for emotional exhaustion (EE), 5 items for depersonalization (DP), and 9 items for personal accomplishment (PA). Each item was answered on a 7-point scale which ranges from never (=0) to daily (=6). The total scores of each dimension are summed up and categorized into low, moderate, or high. Cut-off points of professional burnout scores were as follows: emotional exhaustion: low (≤16), moderate (17-26), and high (≥27); depersonalization: low (≤6), moderate (7-12), and high (≥13); and personal accomplishment: low (≤31), moderate (32-38), and high (≥39). A nurse is considered to be in burnout when exhibiting high levels of emotional exhaustion and/or depersonalization and low regarding personal accomplishment [20].

### 2.9. Risk of Bias in Individual Studies

Methodological quality and risk of bias assessments were performed by two reviewers (AH and WA), blindly and independently. The blinding was maintained by using the Covidence software that allows/obligates each reviewer to work without knowing the other reviewer's choice. This diminishes errors and the risk of bias in the selection of the studies. Disagreements were solved by discussion and where necessary by arbitration involving a third reviewer/author.

### 2.10. Data Analysis and Presentation of Results

Data were analyzed using the R and RStudio software. Forest plots were drawn to visualize the combined prevalence of burnout and the extent of statistical heterogeneity between studies. Statistical heterogeneity was assessed using the *χ*^2^ test on Cochran's *Q* statistic 20 and quantified by calculating the *I*^2^ statistic (with values of 25%, 50%, and 75% and above representative of a low, medium, and high heterogeneity, respectively). There was heterogeneity between studies included in this study. Consequently, we used a random-effects meta-analysis to estimate the overall pooled prevalence of burnout; this might be due to variation in the study area and the difference in sample size. To assess possible publication bias, funnel plot test methods were used.

### 2.11. Data Synthesis

This meta-analysis was performed to estimate the pooled prevalence of burnout among nurses in Ethiopia. Results were presented using forest plots. Variation due to a small number of studies plays a great role on the pooled prevalence of burnout to occur by chance. A subgroup analysis was summarized by geographic regions where the study was conducted because there was heterogeneity between the studies. A random-effects meta-analysis was performed [21] to determine the pooled estimate of the prevalence of burnout in Ethiopia. Heterogeneity was explored using Cochran's *Q* and quantified by *I*^2^ statistics [22]. A sensitivity analysis, removing 1 study from the analysis at a time, was done to ensure that none of the studies included in the meta-analysis produced significant variations in the mean prevalence rates obtained. Results were reported as proportions with corresponding 95% confidence intervals (CIs). The results of this review were reported based on the Preferred Reporting Items for Systematic Reviews and Meta-Analyses (PRISMA) guidelines [23] and registered in the PROSPERO database with ID number CRD42020188092.

## 3. Results

### 3.1. Screening Flow

**Figure 1:**
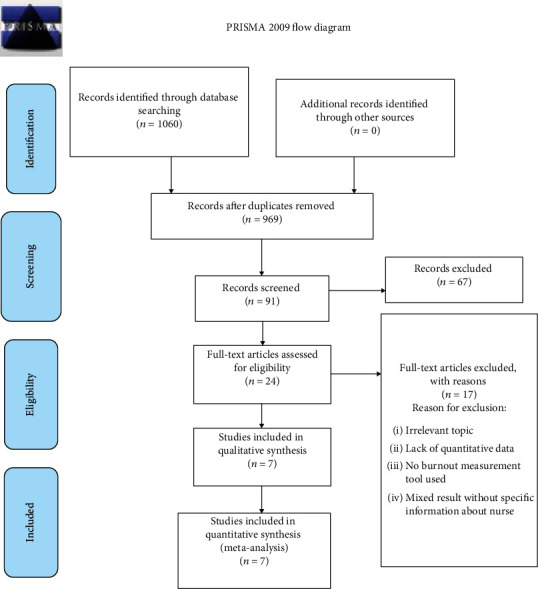
Flow chart diagram showing the selection of articles for systematic review and meta-analysis of burnout among nurses in Ethiopia 2020.

Initially, we have selected 1060 studies for evaluation, and then, irrelevant studies (duplicate studies (969 studies), studies not related to the study topic, and studies that did not include nurses (326 studies)) were excluded from the review. We have included 24 documents for full-text reading. Finally, seven studies were included in the review after applying inclusion and exclusion criteria (Figure 1 and Table 1).

### 3.2. Study Characteristics

In the meta-analysis, seven studies were included. Three (42.8%) of the studies were from Amhara region [24–26], and the four studies were from Addis Ababa, Oromia, SNNPR, and Tigray [14–17]. All of the studies were cross-sectional studies [14, 15, 17, 24–26] except one study which was cohort study design [16]. Of all the studies, three of them were conducted among nurses only [17, 24, 26], and the rest were conducted among healthcare professionals [14–16, 25]. The total sample size for this review was 1654 nurses with a maximum sample of 369 from Amhara region [24] and a minimum sample of 75 from SNNPR [16]. The quality score of each primary study, based on the Newcastle-Ottawa Scale quality assessment criteria, showed no considerable risk; therefore, all the studies were considered in this systematic review and meta-analysis (Table 2).

### 3.3. The Pooled Prevalence of Burnout

**Figure 2:**
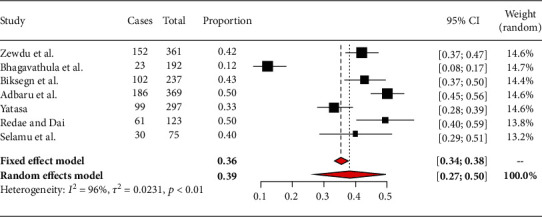
Forest plot for the pooled prevalence of burnout among nurses in Ethiopia from seven observational studies, 2020.

In this review, the overall pooled prevalence of burnout among nurses in Ethiopia was 39% (95% CI: 27% to 50%). The heterogeneity test was checked in this analysis, and it was *I*^2^ = 96% indicating that there is heterogeneity (Figure 2).

### 3.4. Subgroup Analysis of Burnout among Nurses by Region of the Country

**Figure 3:**
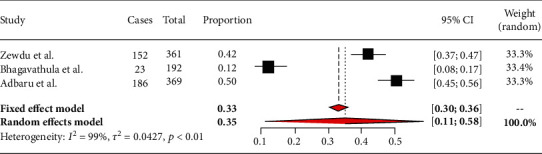
Forest plot showing burnout among nurses in Amhara region.

There are three studies done in Amhara region, but the other regions had one and no studies at all. Based on the subgroup analysis, burnout among nurses attending Amhara region hospitals was 35% (95% CI: 11%-58%) (Figure 3). In other regions, the prevalence of burnout among nurses was as follows: Oromiya 43% (95% CI: 37%-50%), Tigray 50% (95% CI: 40%-59%), Addis Ababa 33% (95% CI: 28%-39%), and SNNPR 40% (95% CI: 29%-52%) [14–17] (Figure 2).

**Figure 4:**
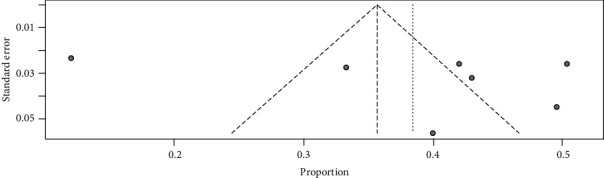
Funnel plot showing evidence of publication bias across the studies.

A funnel test was used to test for publication bias. In this study, the funnel plot showed that there is publication bias (Figure 4).

## 4. Discussion

The present systematic review with meta-analysis was performed to produce pooled estimates of the nationwide prevalence of burnout among nurses in Ethiopia. The emphasis of this review was to assess the burden of burnout among nurses in Ethiopia for a better understanding of the condition and to prevent the problem of burnout throughout the country.

The pooled prevalence of burnout in this review was 39% (95% CI: 27% to 50%). Individually, the prevalence of burnout among nurses across the regions in Ethiopia ranges from 12% in Amhara [27] to 50.6% in Oromia [14]. The reason for those variations might be due to the difference in the study setting because two of the studies were conducted in hospitals and health centers, while five of the studies were conducted in hospitals only; this difference may result in variation in the prevalence of burnout. Another possible factor for this variation might be due to the study population because four of the studies were conducted among healthcare providers but they have reported the prevalence of burnout among nurses in Ethiopia, while three of the studies were conducted among nurses only (Table 1).

The result of this review is consistent with the systematic review done worldwide; the overall prevalence of burnout among pediatric nurses was 31% [28] and among emergency nurses was 30% [29]. It is also comparable with the study conducted in Iran; the overall prevalence of burnout among Iranian nurses was 36% [30]. Similarly, our finding was also consistent with the study done in sub-Saharan Africa which was 33% [9].

The prevalence of this review is higher than the systematic review done globally; the overall pooled prevalence of burnout among global nurses was 11.23%. There are supportive evidence that shows there is a significant difference noted between geographical regions, specialties, and type of burnout measurement used. The sub-Saharan African region had the highest burnout prevalence rate while Europe and Central Asia region had the lowest prevalence [7]. The possible difference could be due to the fact that this review was done in one of the sub-Saharan countries with the highest prevalence of burnout among nurses in the world [7]. This could be the reason for the highest prevalence in this review.

On systematic review of the studies included in this review, gender (female sex), job insecurity, working environment, low interest in the profession, night shift, long hour workload, work experience, health status, and intention to leave current work are the major factors associated with burnout among nurses [14–16, 25]. This review is similar to the systematic review done in sub-Saharan Africa wherein workload, interpersonal and professional conflict, emotional distress, and low social support are the factors associated with burnout among nurses [31]. It is also comparable to a systematic review done in seven Arabian countries in which gender, nationality, service duration working hours, and shift pattern are the major factors associated with burnout among nurses [32].

This review is the first review to synthesize published studies and to estimate pooled prevalence of burnout among nurses in Ethiopia. The finding of this review suggests that nurses have high burnout prevalence warranting attention and implementation. This study will serve as an input for interventional studies, for policy improvement in nurses' work conditions and overall healthcare quality.

### 4.1. The Relevance of This Study for Clinical Practice

This review shows that the prevalence of burnout among nurses is high. This high prevalence has an effect in the quality of nursing care practice, patient quality of care, and overall healthcare system. Reducing this prevalence will have a great contribution to the quality of clinical practice and the health status of nurses and the patients especially in developing countries.

### 4.2. Implications of This Study for Nursing Management

This systematic review contributes valuable information concerning burnout among nurses in Ethiopia. It is very important to the scientific community as a whole, but particularly to nurses working in nursing administration and policy. This result shows that the prevalence of burnout is high which suggests that a significant number of nurses are either affected by burnout or liable to develop soon. Thus, nursing management should prepare an attractive environment for nurses to reduce the impact of burnout on the profession and the overall healthcare system.

### 4.3. Implications of This Study for Policy and Practice

The results we present show that interventions should be undertaken to reduce burnout among nurses. Thus, providing better workplace conditions and the introduction of interventions which are suitable for nurses have a crucial positive effect on the quality of care provided and on patient satisfaction.

### 4.4. The Implication of This Study for Researchers

The finding of this review will serve as a source of information on the burden of burnout among nurses in Ethiopia. Further longitudinal studies should be conducted, focusing on burnout risk factors and measures to attenuate the symptoms observed and coping strategies.

### 4.5. Limitations of This Study

The number of studies included in this review is small that is why it is difficult to do metaregression for the associated factors of burnout. The few numbers of studies included affected the result of the pooled prevalence of burnout by affecting the precision. Variation in studies plays a great role in the pooled prevalence of burnout to occur by chance. This meta-analysis did not present more detailed data on these three dimensions of burnout (depersonalization, personal accomplishment, and emotional exhaustion) and possible risk factors because there is no enough data from the included studies and also there is no enough information to do subgroup analysis based on subspecialization, department, unit, and/or ward.

## 5. Conclusions

Burnout affects two out of five nurses in Ethiopia. Therefore, effective interventions and strategies are required to reduce burnout among nurses. The finding of this review will serve as a source of information on the burden of burnout among nurses for clinical nurses, academic nurses, administrative nurses, and researcher nurses. Finally, this finding is very important not only for nurses but also for the policymakers, program planners, and other researchers which will be used as evidence for implementing appropriate interventions and implementations to decrease the prevalence of burnout among nurses in Ethiopia.

## Figures and Tables

**Table 1:** Search terms and strategy for burnout among nurses in Ethiopia.

Sr. No.	Databases	Number of articles found	Number of articles included	Number of articles excluded
1	PubMed/MEDLINE	*N* = 106	*N* = 7	*N* = 99
2	Google Scholar	*N* = 448	*N* = 7	*N* = 441
3	Web of Science	*N* = 68	*N* = 2	*N* = 66
4	Cochrane Library	*N* = 173	*N* = 6	*N* = 167
5	Africa-Wide Information	*N* = 167	*N* = 7	*N* = 160
6	African Index Medicus	*N* = 98	*N* = 7	*N* = 91
7	Total	*N* = 1060	*N* = 36 but the included studies were 7 studies because there is duplication	*N* = 1024

NB. 969 articles were duplicated in the database information, and 67 articles were excluded due to irrelevant topics, lack of quantitative/prevalence data, no burnout measurement tool used, and studies done on healthcare providers but there is no specific result about the prevalence of burnout among nurses independently reported.

**Table 2:** Characteristics of studies included in the systematic review and meta-analysis of the prevalence of burnout among nurses in Ethiopia.

Authors and year of publication	Study region	Data collection year	Study design	Sample size	Cases	Prevalence (%)	Study population	Study setting	Diagnostic criteria of burnout	Quality assessment (based on NOS)
Zewdu et al., 2017 [26]	Amhara	February 20 to March 30/2017	CS	361	152	42.10	Nurses	Mixed^∗^	MBI-HSS	8
Bhagavathula et al., 2018 [27]	Amhara	September to November 2016	CS	192	23	11.98	HCP	Hospital	MBI-HSS	7
Biksegn et al., 2016 [14]	Oromiya	November to December 2013	CS	237	102	43.03	HCP	Hospital	MBI-HSS	8
Adbaru et al., 2019 [24]	Amhara	March to April 2017	CS	369	186	50.40	Nurses	Hospital	MBI-HSS	9
Yatasa, 2014 [17]	Addis Ababa	June to December 2013	CS	297	99	33.33	Nurses	Hospital	MBI-HSS	9
Redae and Dai, 2019 [15]	Tigray	March to April 2017	CS	123	61	49.60	HCP	Hospital	MBI-HSS	7
Selamu et al., 2019 [16]	SNNPR	July to December 2014	CS	75	30	40.00	HCP	Mixed	MBI-HSS	6

SNNPR: Southern Nation Nationalities and People Representative; CS: cross-sectional; CO: cohort; HCP: healthcare professionals; NOS: Newcastle-Ottawa Scale; MBI-HSS: Maslach's Burnout Inventory-Human Services Survey; ^∗^mixed: it includes hospital, health center, and/or health post.

## Data Availability

The data analyzed during the current meta-analysis is available from the corresponding author on a reasonable request.

## Abbreviations

PRISMA-P: Preferred Reporting Items for Systematic Review and Meta-Analysis Protocols
SNNPR: Southern Nation Nationalities and People Representative
CI: Confidence interval.
